# Formability and Electromagnetic Performance Comparison of Fe-P-Based SMC and Fe-5.0 wt.%Si Powders

**DOI:** 10.3390/ma18184405

**Published:** 2025-09-21

**Authors:** Seongsu Kang, Seonbong Lee

**Affiliations:** 1 Department of Mechanical Engineering, Keimyung University, Daegu 42601, Republic of Korea; 5472068@stu.kmu.ac.kr; 2 Department of Automotive Engineering, Keimyung University, Daegu 42601, Republic of Korea

**Keywords:** motor, soft magnetic composites (SMCs), powder metallurgy (PM), microstructure, morphology

## Abstract

This study investigates the comparative applicability of Somaloy 700HR 5P and Fe-5.0 wt.%Si powders for axial flux permanent magnet (AFPM) motor cores in low-speed electric vehicles. Optimal forming conditions were derived through Taguchi-based simulations, considering corner radius, forming temperature, and forming speed, followed by prototype fabrication and validation. Simulation and SEM-EDS analyses confirmed consistent density distribution trends, and XRD verified phase stability during forming. While Fe-5.0 wt.%Si exhibited ~10% ± 2 superior electromagnetic performance in the powder state, its motor dynamo performance decreased by 19–25% (*n* = 1) compared to Somaloy 700HR 5P. This discrepancy was attributed to its ~4% lower target density (7.19 ± 0.02 g/cm^3^ vs. 7.51 ± 0.01 g/cm^3^, *n* = 3), assembly-induced mechanical losses, and non-uniform insulation layer caused by residual H_3_PO_4_ and Mo segregation. Somaloy 700HR 5P, despite a higher relative density variation (0.084 ± 0.002 g/cm^3^ vs. 0.063 ± 0.003 g/cm^3^ for Fe-5.0 wt.%Si), achieved an average density close to 7.5 g/cm^3^ and delivered more stable motor performance. Overall, Somaloy 700HR 5P was identified as a more suitable candidate for AFPM motor cores in low-speed EV applications, balancing formability and electromagnetic performance.

## 1. Introduction

Recently, the eco-friendly automobile industry has been urging a shift toward a high-efficiency, low-cost sector. In response to this change, the demand for electric vehicles, hybrid vehicles, and hydrogen fuel cell vehicles has surged dramatically compared to traditional internal combustion engine vehicles. This is because eco-friendly vehicles use environmentally friendly fuels that replace fossil fuels. Eco-friendly vehicles use electromagnetic motors as their main power source, and the electromagnetic core is a key component of these electromagnetic motors [1,2,3,4].

Motors using electromagnetic cores have been continuously used across various industries. In the traditional automotive market, electromagnetic motors primarily use rotors and stators made from laminated electrical steel sheets. Laminated cores offer advantages such as cost-effective manufacturing processes and stable electromagnetic properties. However, electromagnetic motors applied in eco-friendly vehicles require higher efficiency and precision, and laminated cores have limitations in meeting these demands. Additionally, to minimize energy loss, it is necessary to reduce the thickness of the steel sheets, which raises challenges related to mechanical dimensional accuracy in the lamination direction and environmental pollution caused by scrap remaining after shaping. To overcome these limitations, there is a rapidly growing demand in the eco-friendly automobile market for alternative materials to electrical steel sheets.

Soft Magnetic Composites (SMCs) are materials composed of iron-based particles coated with an insulating layer and are emerging as next-generation materials to replace laminated electrical steel sheets [5,6]. Compared to laminated steel sheets, SMCs offer several advantages, including three-dimensional magnetic circuit design freedom, lower core losses, and minimized material waste. Using SMCs enables the production of motors with complex shapes, such as Axial Flux Permanent Magnet (AFPM) motors, which are impossible to manufacture with laminated cores. This design freedom and reduced core loss provide benefits such as motor size reduction and greater flexibility in vehicle design [7,8]. In this context, SMCs are increasingly recognized as advanced materials for electric motor cores. However, the use of SMCs entails trade-offs among core loss, saturation flux density, and formability, necessitating the careful selection of materials optimized for specific applications [9,10,11].

Various prior studies based on SMCs have been conducted to date, among which Fe–Si materials have attracted attention as a leading candidate to replace laminated electrical steel sheets. Recently, active research has been carried out to investigate the magnetic and mechanical properties of Fe–Si alloys across a wide compositional range, from approximately 3.5 wt.% to 9 wt.% [12,13,14,15,16]. The Fe-5.0 wt.%Si powder used in prior research was produced based on a phosphate coating process and demonstrated high permeability and low iron loss characteristics [17]. According to those study results, Fe-5.0 wt.%Si achieved a permeability performance of 90–100 in the 100 kHz frequency range, and it was reported that toroidal cores with a density of approximately 7.42 g/cm^3^ could be manufactured. This study aims to build upon these previous findings by comparing the relative performance with commercially available SMCs currently used in the motor market. In particular, the Somaloy series from Höganäs is a representative product line widely used in the field of SMCs for electrical equipment and motors [18,19]. These SMCs are materials primarily based on pure iron powder coated with an insulating layer to enhance electrical performance. A notable example is the AFPM motor case utilizing Somaloy powder. In prior research by Sim and Lee, formability evaluations of toroidal shapes were conducted using Somaloy 130i 5P and 700HR 5P powders. The results showed that Somaloy 700HR 5P exhibited superior characteristics in terms of stress distribution during forming, leading to its selection as the powder for application in this study [20].

In this study, Fe-5.0 wt.%Si powder and the commercial powder Somaloy 700HR 5P were selected as the comparison materials for AFPM motor applications. While many previous studies have investigated the electromagnetic performance of Fe–Si SMCs in powder or toroidal cores, direct comparisons with commercial powders under AFPM-specific forming and assembly constraints remain scarce. This gap motivated the present work.

This study was designed to test two specific hypotheses:

(i) Fe-5.0 wt.%Si, owing to its higher electrical resistivity and superior electromagnetic performance in the powder and toroidal states, will also demonstrate superior torque and power output in AFPM motor prototypes.
(ii) Somaloy 700HR 5P, due to its higher achievable density, uniform insulation characteristics, and optimized processability, will ensure greater forming stability and more consistent motor performance.

To evaluate these expectations, this study examines bulk density and relative density variation (∆ρ) from compaction experiments and simulations, magnetic losses and flux density at 100–2000 Hz, and torque/power characteristics of AFPM motor prototypes operated at 1000 rpm and 2000 rpm. By integrating these metrics, this work establishes a consistent framework to identify the more suitable candidate material for AFPM motor cores in low-speed EV applications.

Therefore, in this study, we conducted the following:

1. Based on prior research, a comparison group of Fe-Si powder and commercial powder was selected;
2. The target motor for application was chosen and the scope of comparative measurements was established;
3. Experimental conditions and boundary conditions were set according to the applied powder and motor core shape;
4. Process control simulations for motor core manufacturing were conducted based on the target performance of each powder to derive optimal process conditions;
5. Prototypes were produced using the derived optimal process conditions;
6. The microstructure and electromagnetic performance characteristics of the prototypes were comparatively evaluated;
7. Finally, the SMC powder cores will be quantitatively evaluated by applying them to the target motor.

## 2. Materials and Methods

As mentioned in the introduction, among SMCs, Fe-Si powders exhibit a trade-off between formability and electromagnetic performance depending on the Si content [21]. Therefore, in this study, based on previous research that considered the balance between electromagnetic properties and mechanical formability, 5.0 wt.%Si powder was selected as the representative composition. Additionally, to compare the performance of the Fe-5.0 wt.%Si powder, Somaloy 700HR 5P (Höganäs AB, Höganäs, Sweden), a widely used commercial SMC, was chosen as the comparison target.

Additionally, the AFPM motor was selected as the target motor for application. The AFPM motor offers excellent performance relative to its volume due to its high torque density, compact shaft length, and planar core structure, which align well with the shape flexibility and net-shape molding characteristics of SMCs [22]. In particular, utilizing SMCs in the unique axial structure of the AFPM allows for easy design of complex 3D magnetic circuits while simultaneously minimizing iron losses and enhancing manufacturing flexibility.

The specifications of the target motor were set to reflect the driving conditions of a low-speed electric vehicle for testing purposes. This study aimed to apply the motor within a relatively low-speed operating range, up to a maximum of 2000 rpm. The comparative measurement range was divided into two operating conditions. The first condition involved applying a current of 150 A at 1000 rpm, and the second involved applying a current of 130 A at 2000 rpm. These settings can serve as appropriate criteria for verifying the performance of test motors in the low-speed driving range.

### 2.1. Material Properties

To reflect the mechanical and thermal properties of the two powders in the forming simulation, the stress–strain curves, thermal conductivity, and thermal expansion coefficients of Somaloy 700HR 5P and Fe-5.0 wt.%Si powders were applied. The physical properties of Somaloy 700HR 5P were obtained from previous studies and manufacturer-provided data [23], while the values for Fe-5.0 wt.%Si were referenced from reported data in prior research [17].

According to the manufacturer’s datasheet (Höganäs, 2016) [23], the recommended warm-compaction temperature for Somaloy 700HR 5P is 100 °C. In this study, the actual molding temperatures applied were room temperature (RT), 50 °C, and 100 °C. For each condition, both the die and powder were equilibrated at the target temperature for approximately 60 s before pressing to ensure stable warm-compaction conditions. In this study, the optimal molding temperature identified through the toroidal core molding process was approximately 80 °C, which aligns with the manufacturer’s recommended range. In contrast, Fe-5.0 wt.%Si powder requires a molding temperature above 500 °C due to increased mechanical strength resulting from its high silicon content.

The chemical composition and coating composition of the Fe-5.0 wt.%Si powder are summarized in Table 1, citing previous research results. To verify the chemical composition of the comparative material, Somaloy 700HR 5P, XRF (X-ray Fluorescence) analysis was conducted. The analysis was performed using the SEA 1200 VX instrument from Seiko Instruments Inc. (Chiba, Japan), and the results are presented in Table 1 alongside the composition of Fe-5.0 wt.%Si. The analysis confirmed that the Fe-5.0 wt.%Si powder met the designed silicon content of 5.0 wt.%, while Somaloy 700HR 5P exhibited a relatively lower silicon content of 2.74 wt.%. Additionally, as a commercial product, Somaloy 700HR 5P contains additives for insulation; the XRF results showed a phosphate composition of 1.58 wt.% and other additives totaling 1.08 wt.%. Meanwhile, the Fe-5.0 wt.%Si base powder itself does not include insulation or lubrication components, and the additive compositions for these purposes are referenced from previous studies and presented in Table 2.

**Figure 1:**
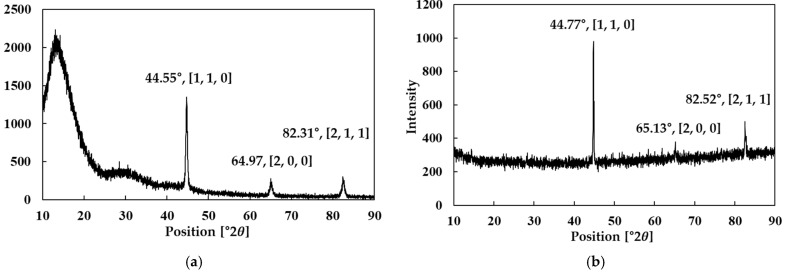
Powder XRD results: (**a**) Somaloy 700HR 5P, (**b**) Fe-5.0 wt.%Si.

In addition, to confirm the phase structure of the two powders in their powder state and to track any unnecessary phase changes during the forming process, the XRD results of the powders reported in previous studies were cited in Figure 1. The analysis showed that both powders exhibited major Bragg peaks at [110], [200], and [211], which correspond to the cubic Fe–Si phase [24]. However, slight shifts in peak positions and differences in relative intensities were observed between Somaloy 700HR 5P and Fe-5.0 wt.% Si. These differences are attributed to the decreased lattice constant caused by the high Si content in Fe-5.0 wt.%Si, which results in the diffraction peaks shifting toward higher angles. In the case of Somaloy 700HR 5P, the variation in peak intensity distribution is interpreted as being influenced by the insulating coating and additives. Such peak shifts and intensity changes have also been reported in previous studies on Fe–Si alloys [25].

**Figure 2:**
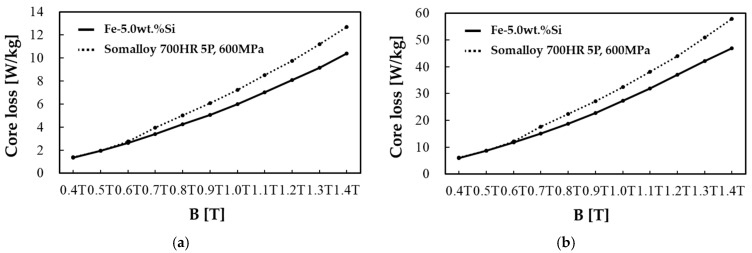

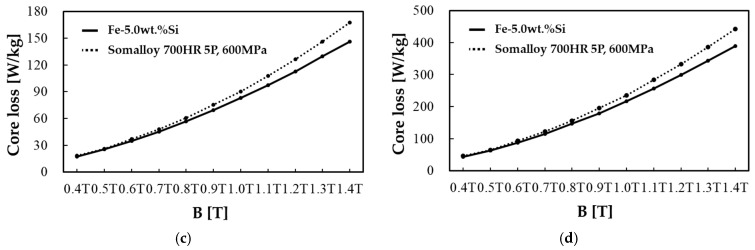
Core loss results of Somalloy 700HR 5P and Fe-5.0 wt.%Si: (**a**) at 100 Hz, (**b**) at 400 Hz, (**c**) at 1000 Hz, (**d**) at 2000 Hz.

**Figure 3:**
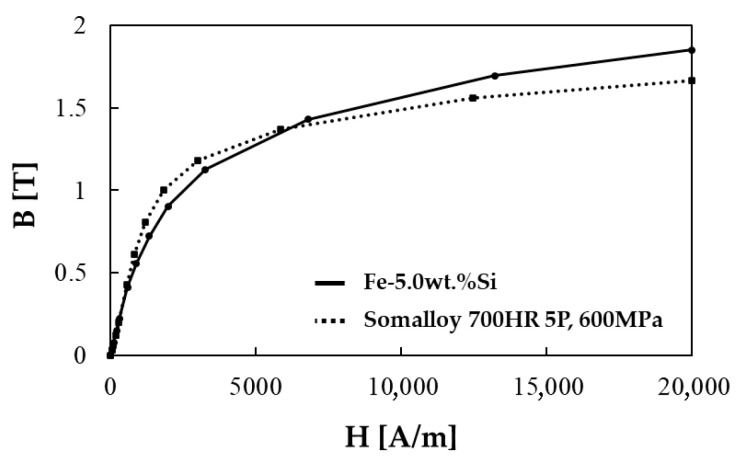
Magnet flux density results of Somalloy 700HR 5P and Fe-5.0 wt.%Si.

Before applying to the motor core shape, an electromagnetic performance evaluation based on a toroidal shape was conducted to compare the basic electromagnetic properties of the two selected SMC powders. Toroidal specimens for both Somaloy 700HR 5P and Fe-5.0 wt.%Si were fabricated to a matched density of approximately 7.5 g/cm^3^ to ensure a fair baseline comparison. Iron loss was measured using the IWATSU SY-8219 (IWATSU Electric Co., Ltd., Tokyo, Japan) with primary and secondary windings applied to the toroidal cores. Iron loss was obtained under sinusoidal AC excitation at 100, 400, 1000, and 2000 Hz with the flux density amplitude controlled (±1%), and the results are shown in Figure 2. Magnetic flux density was measured under a DC magnetic field environment using the Remagraph C-500 (Dr. Förster GmbH & Co. KG, Reutlingen, Germany), where flux density was calculated from the induced secondary voltage and magnetic field strength from the applied current. Since the manufacturer’s provided performance data for iron loss did not include detailed data by magnetic flux density, samples were fabricated using the manufacturing process recommended by the manufacturer and measured under the same conditions as Fe-5.0 wt.%Si. For magnetic flux density, performance data under a molding pressure of 600 MPa for Somaloy 700HR 5P powder, provided by the manufacturer [23], was used to compare with Fe-5.0 wt.%Si. The comparison results of iron loss for Fe-5.0 wt.%Si and Somaloy 700HR 5P at each frequency are presented in Figure 3.

In all measured frequency bands, the iron loss results of the Fe-5.0 wt.%Si specimen showed a reduction of more than 10 ± 2% compared to the Somaloy 700HR 5P powder specimen. Additionally, according to the magnetic flux density results shown in the figure, the Fe-5.0 wt.%Si specimen demonstrates superior magnetic flux density performance around the 6000 A/m. The reason for these results is that Somaloy 700HR 5P powder, being a pure iron-based material, exhibits high magnetic flux density performance in the low-frequency range, but in the high-frequency range and in terms of iron loss, the high resistivity of the Fe-5.0 wt.%Si powder plays a significant role [26]. The fundamental magnetic properties evaluated in this way will be compared with motor performance measured after application to the motor core.

### 2.2. Design of Experiment

To analyze the effects of various process factors and levels, the Design of Experiments (DOE) methodology was applied in this study. When multiple process factors interact simultaneously, as in powder sintering and compression processing, an excessive number of experiments are typically required. Therefore, to systematically analyze the effects of key factors and derive optimal process conditions with a minimal number of experiments, the Taguchi experimental design method was employed [27,28].

Prior to applying the experimental design method for determining optimal process conditions, key factors significantly affecting the process outcomes were preliminarily selected based on prior research. Additionally, the forming speed, which can be directly controlled as a process parameter in actual forming operations, was included as an experimental factor. Since the formability of SMC cores varies depending on their shape, the ‘corner radius’ was added as a factor influencing formability and incorporated into the experimental design [29,30].

In this study, density variation within the specimen was chosen as the characteristic value for evaluating formability. Density variation affects flux uniformity, mechanical strength, and magnetic losses, and is directly linked to core performance. Therefore, density variation was selected as the evaluation metric for formability, and minimizing it was set as a primary objective of the experiments.

The evaluation criterion applied was the ‘smaller-the-better’ function, and the corresponding loss function and Signal-to-Noise Ratio (S/N ratio) are expressed by Equations (1) and (2) as follows.

(1) $${L(y)}_{smaller}=ky^{2}$$

Here, *L*(*y*) quantifies the loss that occurs when the quality characteristic *y* deviates from the target value, and *k* is the quality loss coefficient representing the loss sensitivity. Since this loss function increases proportionally to the square of *y*, the loss is smaller when the measured value is closer to zero and increases sharply as it moves further away. Therefore, this model indicates better quality characteristics when the density deviation is smaller.

(2) $${S/N}_{smaller}=-10\log\;\left[\;\frac{1}{n}\;\sum_{i=1}^{n}{y_{i}}^{2}\right]\;$$

Here, *y_i_* is the quality characteristic value obtained from the *i*-th experiment or analysis, and *n* is the number of repetitions. This formula converts the mean square value of the quality characteristic into a logarithmic scale, where a smaller variance in the values results in a higher signal-to-noise ratio. A higher signal-to-noise ratio indicates greater consistency in the results and that the process is more robust against disturbances. Therefore, in this study, density variation within the specimen was defined as the quality characteristic and analyzed using the smaller-the-better criterion. The signal-to-noise ratios for each level of the process factors were compared and analyzed, leading to the determination of the optimal process conditions.

### 2.3. Experiment Setting

In this experiment, corner radius, secondary forming temperature, and forming speeds of 3, 5, and 7 mm/s were selected as the main control factors affecting density uniformity and internal stress distribution during the forming process. Each factor was set at three levels to reflect actual manufacturing process conditions while quantitatively evaluating the influence of process variables.

The corner radius is a key geometric factor that directly impacts formability and can cause forming non-uniformity such as stress concentration at the corners of the formed part, reduced powder flowability, and localized porosity. Therefore, to analyze the effect of shape sensitivity that may occur during actual forming, three levels of corner radius—0.5 mm, 2.0 mm, and 3.5 mm—considering the core model, were selected as design factor levels.

The secondary forming temperature was selected within the effective forming temperature range derived from the factor influence experiments of prior research. According to previous studies, the condition at 625 °C exhibited the highest density and the most uniform distribution characteristics. Considering process productivity and energy consumption, a range from 450 °C to 650 °C was set at uniform intervals to prioritize the application of the lowest possible temperature conditions. Additionally, for the commercial powder Somaloy 700HR 5P, three temperature levels were selected as design factors: room temperature (RT), 50 °C, and 100 °C, including the manufacturer-recommended forming temperature of 100 °C.

The forming speed was based on the operating range of the forming press equipment and reflected conditions applicable in actual industrial settings. To comprehensively evaluate the effects of forming speed changes on powder flowability, inter-particle friction, and stress transmission characteristics, three levels ranging from 3 to 7 mm/s were selected.

In this study, experiments and evaluations were conducted by applying different forming conditions to two powders (Fe-5.0 wt.%Si and Somaloy 700HR 5P). The reasons for setting different conditions are as follows:

First, the fundamental physical properties of the two powders differ. The forming behavior of SMCs is largely influenced by the powder’s chemical composition and physical properties. According to XRF analysis, both powders are Fe-based SMCs; however, Fe-5.0 wt.%Si has a high silicon content, which is advantageous for electrical resistivity but increases brittleness during forming. Additionally, since each powder has different insulating coatings and lubricant additive compositions, it is necessary to individually set forming conditions suitable for each.

Second, the goal is to derive the optimal forming conditions for each powder and clearly evaluate the effects of process variables. Prior research indicates that the composition of SMCs critically affects performance. Therefore, this study establishes optimal processes reflecting the powder characteristics for each material and quantitatively compares their electromagnetic performance based on these conditions. This approach enables a more reliable evaluation of the performance each material can demonstrate when applied to motor cores.

For these reasons, although the forming factors are the same for both powders, different factor levels were applied for each powder.

A comprehensive arrangement of experimental factors and levels is shown in Table 3 and Table 4, and this experimental design was used as a basis for process optimization.

Furthermore, this experiment applied Taguchi’s method of experimental design, which effectively analyzes the influence of factors with a minimal number of experiments. The experimental array, consisting of three factors each at three levels, was based on an L_9_(3^3^) orthogonal array. This allowed for the quantitative determination of the main effects of forming process variables on the resulting characteristics. The arrangement and levels of experimental factors are summarized in Table 5.

To ensure the performance of porous materials such as SMCs, achieving a certain level of density is essential. In particular, SMCs tend to have weaker inter-particle bonding compared to conventional metal products due to the insulating layers between particles. Therefore, to secure stable mechanical properties, it is necessary to improve density through the control of molding process parameters. Moreover, not only increasing density but also the uniformity of density and stress distribution within the molded body plays a crucial role in securing magnetic properties and core performance [31,32].

Non-uniform density distribution causes uneven magnetic field distribution inside the core and induces localized self-saturation, which reduces the overall system efficiency. This phenomenon especially leads to increased eddy current losses in the high-frequency range, negatively affecting motor operation by causing output reduction and rotational instability. Additionally, uneven density and stress distribution result in decreased mechanical strength, increasing the risk of failure under high-speed rotation or vibration conditions, and impairing the reproducibility and consistency of the manufacturing process, which can adversely affect production yield and quality control [33,34].

To prevent these issues and design optimal molding conditions, this study conducted molding analysis by setting the maximum–minimum deviation of relative density and stress deviation as key characteristic values.

### 2.4. Simulation Design

#### 2.4.1. Soft Magnetic Powder Compression Molding Process

The soft magnetic powder compression molding process is a key technology for manufacturing high-frequency electromagnetic components. It involves high-pressure molding of powders based on iron (Fe), iron–silicon (Fe-Si), and iron–silicon–aluminum (Fe-Si-Al) to produce core electromagnetic parts [35,36,37,38,39]. The critical aspect of this process lies in the insulating layer formed on the surface of the powder particles, which suppresses eddy current losses while maintaining a high saturation flux density.

This process is broadly divided into four stages: first, the filling stage, where soft magnetic powder is loaded into the mold; second, the compacting stage, where high pressure is applied via upper and lower punches to compress the powder; third, the demolding stage, where the molded compact is separated from the mold; and finally, the heat treatment stage, which enhances the mechanical strength and magnetic properties of the molded compact.

During compression molding, an assembly powder mixture of soft magnetic powder and insulating binder is compressed within the mold to form an intermediate molded body called a green compact. After demolding, this green compact undergoes heat treatment to achieve the final product’s required mechanical strength and magnetic characteristics [40,41,42,43,44,45].

In particular, for Fe-Si, it is possible to produce molded bodies with excellent mechanical strength and magnetic properties by applying a 2P2C (2-Pressing 2-Curing) process rather than simple compression [46]. The 2P2C process consists of primary pressing, annealing, secondary pressing, and final heat treatment. The primary pressing shapes the insulated Fe-Si powder close to the final dimensions and density, using either room temperature or warm molds in the temperature range of around 500 °C. The annealing step serves as an intermediate treatment to promote initial particle bonding and relieve residual stresses in preparation for secondary pressing. The secondary pressing removes residual pores in the initially formed product, making the material denser and achieving the final product dimensions. This secondary pressing is also conducted using warm molds at temperatures above 300 °C. In the final stage, heat treatment completes the bonding between particles and forms a uniform microstructure, optimizing mechanical strength and magnetic properties [47,48].

#### 2.4.2. Finite Element Analysis (FEA) of Porous Materials

The FEA of SMCs focuses primarily on the stage where the powder is compressed within the mold [44]. The powder compression stage refers to the process of compressing the powder filled inside the mold, during which the powder rearranges within the mold, leading to an increase in density. This results in the development of internal stress distribution within the powder compact, and the internal density of the compact exhibits a non-uniform distribution. Such internal density distribution directly affects magnetic properties like permeability and saturation flux density, making it crucial to quantitatively predict and control the internal density distribution of the compact during the process [49,50].

FEA is a numerical method capable of simulating these complex forming behaviors. By dividing the actual shape into small elements and applying the physical properties of the powder, it calculates the forces, deformations, and stresses acting on each element over time, allowing for the prediction of how the compact changes throughout the entire process.

When performing finite element analysis that includes plasticity analysis of porous materials, considerations different from those for typical metallic materials are required. Most conventional metallic materials have a polycrystalline structure and thus exhibit isotropic behavior from a macroscopic perspective. However, for porous materials, it is necessary to additionally consider the porosity within the formed body, which prevents the assumption of the material as a continuum. Therefore, models that can indirectly reflect porosity, such as the Shima-Oyane equation, should be applied to quantitatively account for the influence of porosity on the mechanical properties of porous materials [51]. Furthermore, when there are local density variations caused by powder flow during the forming process, the effect of internal density changes on the stress distribution within the formed body must be more accurately represented. The Shima-Oyane equation is shown below in Equation (3), where $\sigma_{y}$, *D*, $\sigma_{m}$ and *f* represent the material’s yield strength, material characteristic coefficient, material’s mean stress and porosity, respectively [52].

(3) $$\sigma_{eq}={\sigma^{2}}_{y}\left[{\left(1-f\right)}^{2}+2f(1+D){\left(\frac{\sigma_{m}}{\sigma_{eq}}\right)}^{2}\right]$$

According to this, as the porosity increases, the yield strength decreases. In the case of a fully dense material with no pores at all, *f* becomes 0, making $\sigma_{eq}$ equal to $\sigma_{y}$. On the other hand, in materials with pores, since *f* is greater than 0, $\sigma_{eq}$ becomes smaller than $\sigma_{y}$.

#### 2.4.3. Simulation Boundary Conditions

**Figure 4:**
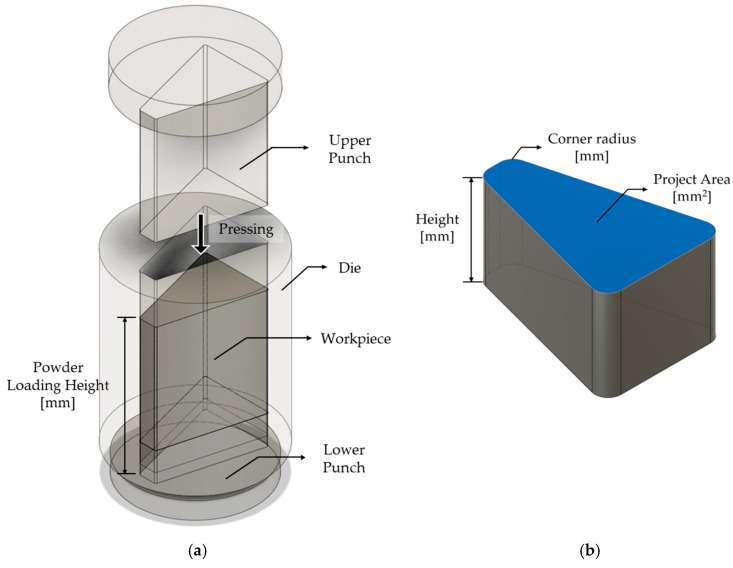
(**a**) Powder compaction process, (**b**) motor stator core geometry.

The powder compaction process and the shape of the motor stator core are shown in Figure 4, with the target height dimension fixed at 14 mm. In this analysis, the powder compaction molding simulation was performed based on this shape.

The initial state of the analysis was set to the green body condition with powder filled, reflecting the actual forming process, and the powder was modeled as a porous structure containing numerous internal pores. The powders applied in the analysis were Somaloy 700HR 5P and Fe-5.0 wt.%Si. The true density of both powders was assumed to be 7.8 g/cm^3^, and the measured tap densities were 3.3 g/cm^3^ and 3.2 g/cm^3^, respectively. Accordingly, the initial relative densities in the analysis were calculated to be approximately 42.3% and 41%.

The target density after final forming was set to 7.3 g/cm^3^ for Fe-5.0 wt.%Si, which corresponds to about 93.59% relative density based on the true density. In other words, the powder undergoes approximately a 2.28-fold increase in density during the forming process, which implies a high-pressure compaction environment accompanied by about 56% shrinkage in volume. Under these conditions, Somaloy 700HR 5P was applied in the study with a target density of 7.5 g/cm^3^, as recommended by the manufacturer.

Such a high compression ratio can directly affect the structural stability of the insulating coating layer formed on the powder particle surfaces. In fact, as the gaps between particles decrease, there is a possibility that the insulating layer may be partially damaged or its continuity may deteriorate. Therefore, to ensure the correlation between the analysis and experimental results and to verify whether the insulation performance is maintained after forming and heat treatment, microstructural analysis using SEM was conducted.

The shape of the stator core includes corner edge curvature radii, and in this study, to evaluate the sensitivity of the shape to forming, three levels of edge curvature radius—0.5 mm, 2.0 mm, and 3.5 mm—were incorporated into the analysis. As the edge curvature radius increases, the effective cross-sectional area projected from the top becomes relatively smaller. Therefore, when filling the same mass of powder, a larger edge curvature radius requires a greater height for the powder to occupy. This affects not only the filling density but also the volume distribution in the initial state and the compression behavior during subsequent forming stages.

Powder forming analysis requires determining the initial filling volume of the powder, which is calculated considering the relative density of the final target density. In this study, the powder charge amount was fixed, and the initial filling volume was set differently according to the projected area for each edge curvature radius level.

To reflect these shape differences, the analysis calculated the top projected area for each edge curvature radius condition and determined the initial filling height corresponding to the initial volume as shown in Equation (4):

(4) $$h_{initial}=\frac{V_{initial}}{A_{round}}$$

Here, *V_initial_* is the initial volume of each powder based on the relative density of the powder, and *A_round_* is the projected area that varies according to the corner radius dimension. According to this relationship, as the corner radius dimension increases, *A_round_* decreases, and consequently, *V_initial_* tends to increase. Based on these calculations, the boundary conditions for the initial filling state under each condition are presented in Table 6. This approach reflects the initial state more closely to the actual geometry, ensuring geometric consistency in the analysis and enabling a quantitative evaluation of density non-uniformity and stress concentration phenomena that may occur during molding.

The finite element analysis model used in this study was designed to reflect the structure of a forming press under uniaxial pressing conditions. A load of 78.5 MPa was applied to the upper punch, and the powder fill height was set as the initial condition of the analysis using the relative density conversion value calculated based on the apparent density.

SMCs generally include wax-based lubricants to reduce friction with the mold during powder forming. This is because the dispersion behavior of the lubricant caused by forming pressure and heat during the molding process affects the powder’s flowability and density uniformity. Accordingly, a friction coefficient of 0.12 was applied between the powder and the mold wall in the boundary conditions of the analysis. For compositions containing MoS_2_-based solid lubricants, friction characteristics can vary depending on the lubricant concentration, powder surface condition, forming speed, and environmental conditions. According to previous studies, the friction coefficient typically ranges broadly from about 0.04 to 0.20 in analysis environments. In this study, considering the convergence of theoretical analysis and reproducibility of actual process conditions, a mid-range value of 0.12 was selected as the friction coefficient. This value is considered to reproduce relatively stable friction behavior under typical conditions where the solid lubricant layer is maintained [53,54].

**Figure 5:**
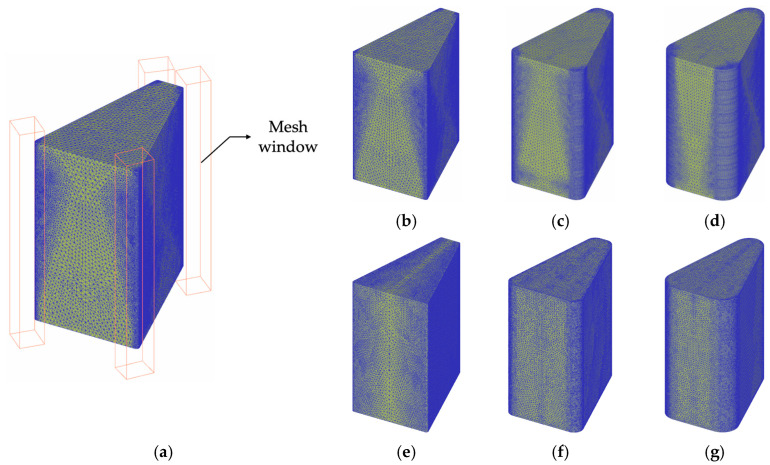
(**a**) mesh window, Somaloy 700HR 5P mesh feature: corner radius (**b**) 0.5, (**c**) 2.0 (**d**) 3.5, Fe-5.0 wt.%Si mesh feature: corner radius (**e**) 0.5, (**f**) 2.0, (**g**) 3.5.

The fixed core model used in the analysis was composed of tetrahedral elements, and a mesh window with a dense mesh was placed in the curvature radius area to effectively analyze the influence of curvature radius on the model. The number of elements for each edge curvature radius is shown in Table 7, and the mesh window layout and element shapes for each model are presented in Figure 5. The analysis was performed using DEFORM-3D (SFTC, version 14.2, Columbus, OH, USA; https://www.deform.com), through which powder flow behavior, density distribution, and internal stress states were analyzed to evaluate the effects of various process parameters.

To further ensure the reliability of the numerical results, additional convergence checks were performed. A mesh refinement study demonstrated that both density and stress predictions became stable once the element count exceeded ~75% of the fully refined mesh (~454k elements). In addition, sensitivity to displacement-step increments (0.015–0.06 mm) was negligible, with variations of less than 0.5%. The detailed results of these verification studies are provided in Appendix A.

### 2.5. Powder Compaction and Heat Treatment Procedures

Both Somaloy 700HR 5P and Fe-5.0 wt.%Si powders were compacted under an Ar atmosphere to prevent oxidation, followed by natural cooling in still air after pressing. After compaction, all specimens underwent heat treatment at 650 °C for 1 h under a nitrogen atmosphere to relieve residual stresses and stabilize the insulation layer.

Separate from the Somaloy 700HR 5P produced through a single compression-heat treatment process, the Fe-5.0 wt.%Si was analyzed by dividing the entire simulation into three stages to replicate the two-step compression molding process used in the actual experiment.

The first stage is the primary forming step, where both the mold and powder were equilibrated at 100 °C for 60 s in Ar atmosphere, and the forming speed is applied according to the experimental design parameters. In this stage, the powder is preliminarily formed by compressing only up to an intermediate height of 15 mm, rather than reaching the total forming height of 14 mm.

The second stage is the temperature ramp-up phase, during which the compact was heated at ~10 °C/s under Ar to the designated secondary forming temperature (450, 550, or 650 °C), followed by a 60 s soak to ensure thermal uniformity. This step reflects the preheating and heat diffusion conditions of the actual forming process and simulates the thermal changes occurring inside the powder.

Finally, the third stage is the secondary forming process, where the preformed compact is compressed again. This step is set to achieve the final target forming height of 14 mm. The forming speed in this stage also follows the experimental design conditions, and the temperature and friction conditions maintain the thermal history from the second stage during compression.

After each stage, specimens were naturally cooled to room temperature in still air. By dividing the process into multiple stages in this way, the simulation effectively incorporated thermo-mechanical coupled conditions and accurately reflected the boundary conditions of the actual 2P2C process.

By dividing the process into multiple stages in this way, the simulation effectively incorporates thermo-mechanical coupled conditions during forming and accurately reflects physical boundary conditions similar to those in the actual process.

## 3. Simulation Results

Through powder compaction molding analysis conducted according to the experimental design, the density and stress distribution characteristics for each process condition in the manufacturing of SMC stator cores were derived. This simulation was performed for each powder type based on a total of nine conditions, consisting of combinations of three predefined key factors: corner radius dimension, secondary molding temperature, and molding speed. Various variables related to relative density and stress were analyzed for each condition.

### 3.1. Somaloy 700HR 5P Results

**Figure 6:**
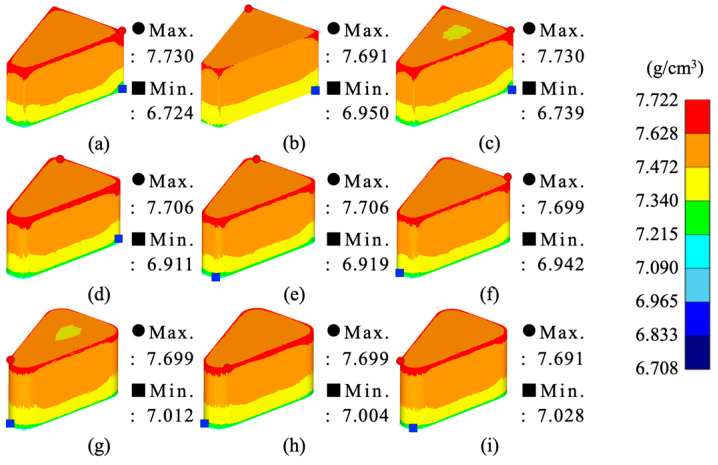
Somaloy 700 HR 5P density results: (**a**) case 1, (**b**) case 2, (**c**) case 3, (**d**) case 4, (**e**) case 5, (**f**) case 6, (**g**) case 7, (**h**) case 8, (**i**) case 9.

**Figure 7:**
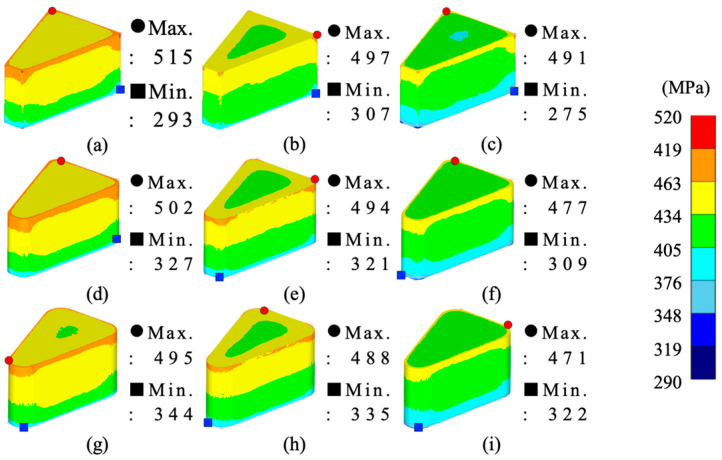
Somaloy 700 HR 5P effective stress results: (**a**) case 1, (**b**) case 2, (**c**) case 3, (**d**) case 4, (**e**) case 5, (**f**) case 6, (**g**) case 7, (**h**) case 8, (**i**) case 9.

**Figure 8:**
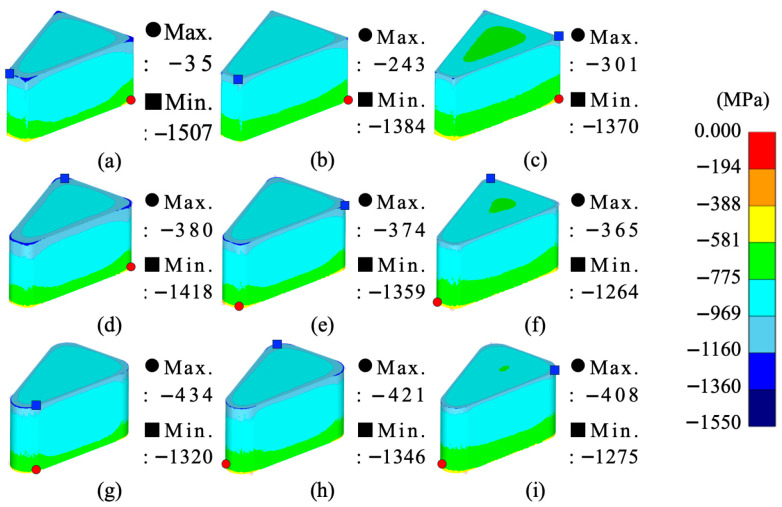
Somaloy 700 HR 5P hydrostatic stress results: (**a**) case 1, (**b**) case 2, (**c**) case 3, (**d**) case 4, (**e**) case 5, (**f**) case 6, (**g**) case 7, (**h**) case 8, (**i**) case 9.

From the simulation results, the maximum–minimum density variation in Somaloy 700HR 5P, average effective stress, average hydrostatic pressure was calculated, and the related values are summarized in Table 8. The density distribution for each condition is visualized in Figure 6, the effective stress distribution in Figure 7, and the average stress distribution in Figure 8, respectively.

Figure 6 visually presents the density distribution of the molded parts under each experimental case. It can be observed that, across all conditions, the highest density is concentrated at the upper part of the molded part, while the lowest density is focused in the lower corner areas. This is analyzed to be due to the high velocity and strain rate acting on the upper part during the downward movement of the upper punch, while powder flowability decreases and friction concentrates at the lower mold and corner regions.

Additionally, the density variation for each analysis case is shown. Case 9 recorded the lowest variation at 0.663 g/cm^3^, which is an improvement of over 15% compared to the overall average, indicating the best density uniformity. This case consists of a corner curvature radius of 3.5 mm, a molding temperature of 100 °C, and a molding speed of 5 mm/s. The reasons for the minimal density variation under this case are as follows: first, a larger curvature radius reduces stress concentration at the corners [55], and second, low-speed molding gradually compresses the powder without sudden load changes, reducing density non-uniformity [56]. Additionally, the 100 °C condition provides sufficient powder flowability compared to room temperature and 50 °C, and lowers the stress level required for plastic deformation of the metal.

Figure 7 shows the distribution of effective stress inside the formed part under each simulation case. In all cases, the maximum effective stress was concentrated at the upper corners of the formed part or areas with sharp curvature, while the minimum stress was observed on the lower wall surface. This is considered a typical stress distribution pattern caused by stress concentration due to the downward movement of the upper punch, as well as flow resistance and amplified friction in the corner regions.

The maximum value of effective stress variation from the analysis results was highest at 222.527 MPa under analysis case 1. This appears to be due to stress concentration effects caused by the relatively small radius of curvature and forming temperature. In contrast, under analysis cases with larger radii of curvature, the effective stress variation decreased by an average of 22% at a radius of curvature of 2.0 mm and by 32% at 3.5 mm compared to the maximum value. Notably, analysis case 9 showed the minimum effective stress variation, with a stress variation approximately 33% lower than the maximum value.

Figure 8 visually shows the distribution of hydrostatic stress in each simulation case. The average stress is negative in all conditions, indicating an isotropic compressive stress state typical of a compression molding environment.

The location of the maximum hydrostatic pressure concentration is at the lower corner of the molded part, which is believed to be due to the compressive forces during molding concentrating in areas where contact with the mold and resistance to powder flow are highest.

In simulation cases 3, 6, and 9, where the molding temperature is 100 °C, the hydrostatic pressure level is approximately −802 MPa, showing a similar level and representing the minimum pressure conditions during molding. On the other hand, simulation cases 1, 4, and 7, with room temperature molding, exhibit the highest hydrostatic pressure level of about −850 MPa, indicating conditions with a higher likelihood of internal residual stress generation and reduced mold durability.

Across all nine simulation cases, the hydrostatic pressure level showed a strong dependence on the molding temperature, exhibiting a clear decreasing trend with increasing temperature, while the radius of curvature and molding speed do not appear to have a significant effect on changes in hydrostatic pressure.

**Figure 9:**
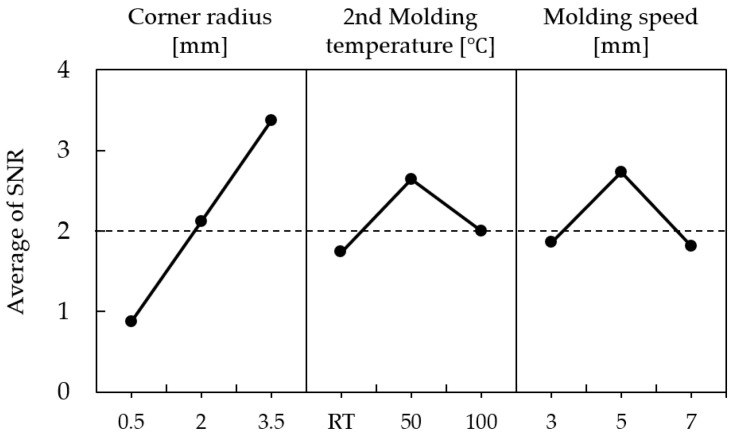
Average S/N ratio of control factor for Somaloy 700HR 5P.

The maximum and minimum values of density, selected as characteristic values of the simulation, were applied as the “smaller-the-better” characteristic for calculating the signal-to-noise ratio. The signal-to-noise ratio values, calculated based on the relative density differences for each analysis case, are summarized in Table 9, with analysis case 9 showing the highest S/N ratio. The main effects of the S/N ratio for each level of the control factors are organized in Table 10 and Figure 9.

The factors affecting the level of density variation were found to be, in order of significance, edge curvature radius, secondary molding temperature, and molding speed. According to the main effect analysis of the S/N ratio, the optimal case was determined to be 3.5 mm, 50 °C, and 5 mm/s, respectively. Although the hydrostatic stress analysis indicated that 100 °C reduced compressive stress compared to 50 °C, the Taguchi optimization prioritized density uniformity as the key quality characteristic. Under this multi-factor evaluation, 50 °C provided superior overall formability performance by minimizing density variation. Furthermore, prototype specimens fabricated under the S/N-derived optimum (3.5 mm, 50 °C, 5 mm/s) achieved an average measured density of 7.509 g/cm^3^ with only 0.12% error relative to the simulation target, thereby experimentally validating the chosen condition. Since this case did not correspond to any case in the orthogonal array, an estimation calculation was performed to verify the significance of the experiment.

The predicted value for a curvature radius of 3.5 mm, molding temperature of 50 °C, and molding speed of 5 mm/s was calculated using Equation (5) by summing the effects of each factor from the arithmetic mean of the S/N ratio [57,58].

(5) $$\hat{Y_{opt}}=\bar{A_{3}}+\bar{B_{2}}+\bar{C_{2}}-2\bar{T}$$

Here, $\hat{Y_{opt}}$ is the predicted value, *A*_3_ is the main effect of curvature radius at level 3, *B*_2_ is the main effect of molding temperature at level 2, *C*_2_ is the main effect of molding speed at level 2, and *T* is the overall mean of all response values.

To verify the significance of the experiment, the population mean of the S/N ratio at the optimal process conditions was calculated as 4.469 and compared with the predicted S/N ratio of 4.471 calculated using Minitab (Minitab, LLC, State College, PA, USA; https://www.minitab.com, accessed on 11 July 2025) for the optimal process conditions. As a result, the error in the S/N ratio was 0.04%, confirming the validity of the experiment.

### 3.2. Fe-5.0 wt.%Si Results

**Figure 10:**
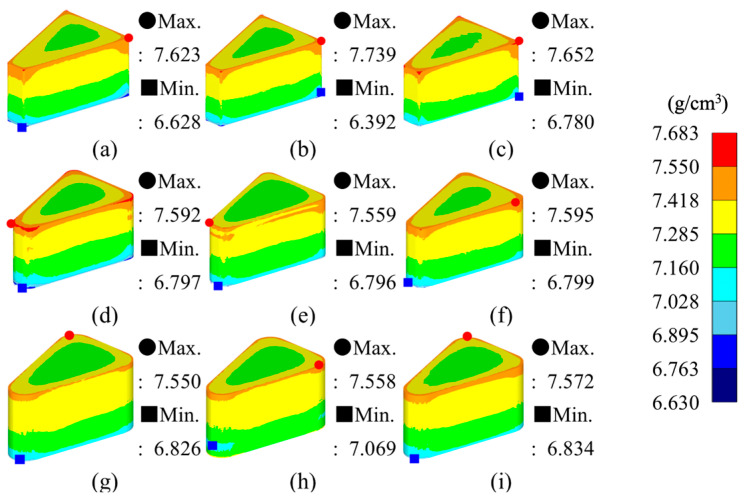
Fe-5.0 wt.%Si density results: (**a**) case 1, (**b**) case 2, (**c**) case 3, (**d**) case 4, (**e**) case 5, (**f**) case 6, (**g**) case 7, (**h**) case 8, (**i**) case 9.

**Figure 11:**
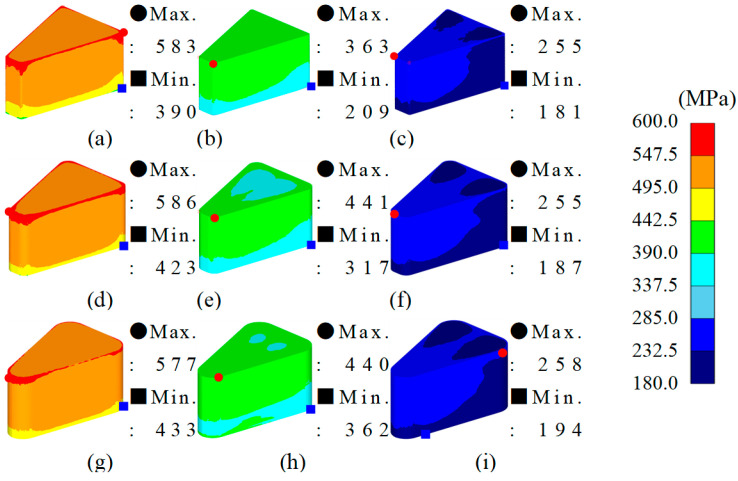
Fe-5.0 wt.%Si effective stress results: (**a**) case 1, (**b**) case 2, (**c**) case 3, (**d**) case 4, (**e**) case 5, (**f**) case 6, (**g**) case 7, (**h**) case 8, (**i**) case 9.

**Figure 12:**
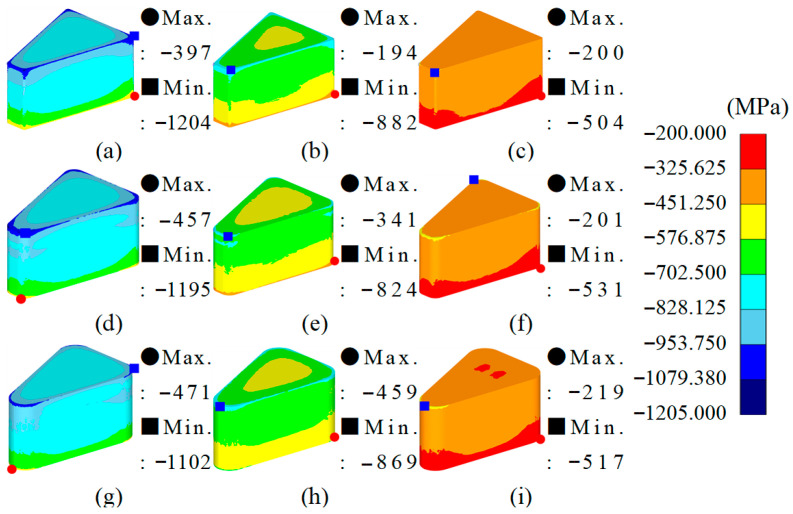
Fe-5.0 wt.%Si hydrostatic stress results: (**a**) case 1, (**b**) case 2, (**c**) case 3, (**d**) case 4, (**e**) case 5, (**f**) case 6, (**g**) case 7, (**h**) case 8, (**i**) case 9.

From the simulation analysis results conducted with Somlaoy 700HR 5P, the maximum–minimum density deviation, average effective stress, average hydrostatic pressure, and upper punch load for Fe-5.0 wt.%Si were obtained. The related values are summarized in Table 11. The density distribution for each case is visualized in Figure 10, the effective stress distribution in Figure 11, and the hydrostatic stress distribution in Figure 12, respectively.

Figure 10 visually presents the relative density distribution of the molded parts for each simulation case. In all cases, the highest density was concentrated at the upper part of the molded part, while the lowest density was found in the lower corner areas. This is analyzed to be due to the same reason as with Somaloy 700HR 5P, where high velocity and strain occur at the top, and powder flowability decreases with concentrated friction at the bottom and corners of the mold.

Table 11 shows the density variation (maximum–minimum density) for each simulation case. Simulation case 8 recorded the lowest variation at 0.489 g/cm^3^. This represents an improvement of over 40% compared to the overall average and indicates the best density uniformity. This case consists of a corner radius of 3.5 mm, a molding temperature of 550 °C, and a molding speed of 3 mm/s. These results are attributed to the fact that a larger radius of curvature reduces stress concentration at the corners. Low-speed molding gradually compresses the powder without sudden changes in load, helping to reduce density non-uniformity. Additionally, the 550 °C condition is considered a thermo-mechanical balance point that provides sufficient powder flowability compared to 450 °C while preventing thermal damage to the insulating layer that can occur at 650 °C.

Figure 11 shows the distribution of effective stress inside the formed part for each simulation case. In all cases, the maximum effective stress was concentrated at the upper corners of the formed part or areas with sharp curvature, while the minimum stress was observed at the lower section. This is considered a typical stress distribution pattern caused by stress concentration from the downward movement of the upper punch, as well as increased flow resistance and friction in the corner regions, similar to Somaloy 700HR 5P.

The analysis results indicate that the maximum effective stress deviation was highest in simulation case 1, measuring 193.663 MPa. This is attributed to the stress concentration effect caused by the relatively small radius of curvature and forming temperature.

As the radius of curvature increased, the effective stress deviation decreased linearly. Simulation case 9 showed the lowest stress deviation at 63.893 MPa. This reflects a stable stress deviation due to the combination of a gentle radius of curvature and high temperature forming conditions.

Figure 12 visually shows the distribution of hydrostatic stress in each simulation case.

The location of the maximum compressive stress concentration is at the lower corner of the molded part, which is the same for Somaloy 700HR 5P. This is believed to be because the compressive forces during the molding process are concentrated in areas where contact with the mold and resistance to powder flow are greatest.

The hydrostatic pressure results, similar to those for Somaloy 700HR 5P powder, depend absolutely on the molding temperature. In simulation cases 3, 6, and 9, where the molding temperature is 650 °C, the hydrostatic pressure results showed similar levels. This represents a decrease of approximately 54% compared to a molding temperature of 450 °C and about 38% compared to 550 °C. Therefore, as the molding temperature increases, the compressive forces applied to both the molded part and the mold decreases to a similar extent.

**Figure 13:**
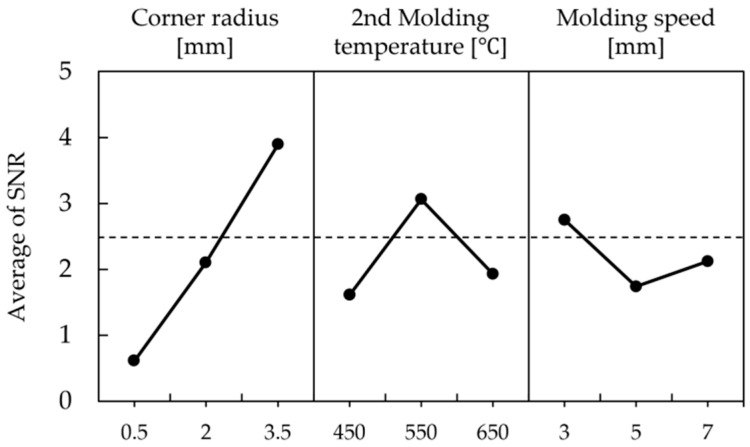
Average S/N ratio of control factor for Fe-5.0 wt.%Si.

The S/N ratio values calculated based on the relative density differences from each simulation result of Fe-5.0 wt.%Si are summarized in Table 12, with simulation case 8 showing the highest S/N ratio value. The main effects of the S/N ratio for each level of the control factors are organized in Table 13 and Figure 13.

The factors affecting the level of density variation were found to be, in order of significance, corner radius, secondary forming temperature, and forming speed. The optimal conditions based on the main effects analysis of the S/N ratio were determined to be 3.5 mm, 550 °C, and 3 mm/s, respectively, which correspond to simulation case 8 that exhibited the highest S/N ratio for magnetic properties. As a result, the conditions set through the design of experiments were confirmed to align with the actual response characteristics, thereby demonstrating the reliability of the Taguchi experimental design.

### 3.3. Result Analysis

**Figure 14:**
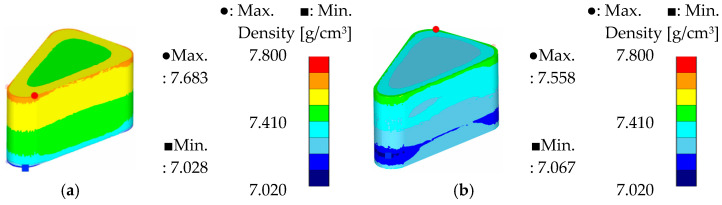
Optimal density results: (**a**) Somaloy 700HR 5P and (**b**) Fe-5.0 wt.%Si.

**Figure 15:**
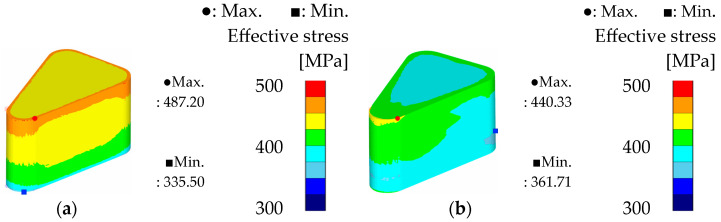
Optimal effective stress results: (**a**) Somaloy 700HR 5P and (**b**) Fe-5.0 wt.%Si.

**Figure 16:**
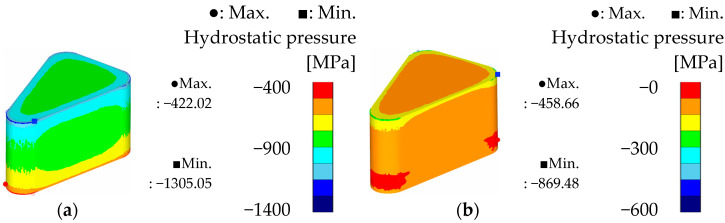
Optimal hydrostatic pressure results: (**a**) Somaloy 700HR 5P and (**b**) Fe-5.0 wt.%Si.

To compare the theoretical formability of the two powders, the simulation results under optimal conditions for each powder are presented in Figure 14, Figure 15 and Figure 16. For Somaloy 700HR 5P, the simulation corresponds to the optimal conditions of a curvature radius of 3.5 mm, forming temperature of 50 °C, and forming speed of 5 mm/s. For Fe-5.0 wt.%Si, the results correspond to simulation case 8.

In the density distribution results shown in Figure 14, Somaloy 700HR 5P achieved an average density closer to the target density of 7.5 g/cm^3^. However, the density deviation was 0.084 g/cm^3^ for Somaloy 700HR 5P and 0.063 g/cm^3^ for Fe-5.0 wt.%Si. Consequently, Fe-5.0 wt.%Si exhibited approximately 25% lower deviation, indicating superior density uniformity.

Figure 15 presents the effective stress results, where the maximum stress for Fe-5.0 wt.%Si was about 10% lower than that of Somaloy 700HR 5P. The average effective stress measured was 431.224 MPa for Somaloy 700HR 5P and 399.009 MPa for Fe-5.0 wt.%Si. Additionally, the stress deviation was 151.705 MPa for Somaloy 700HR 5P and 78.619 MPa for Fe-5.0 wt.%Si, demonstrating that Fe-5.0 wt.%Si formed a more uniform stress distribution. This trend is attributed to the nonlinear behavior where the stress required during powder compaction increases exponentially rather than linearly with increasing density.

In the hydrostatic pressure analysis shown in Figure 16, the deviation for Fe-5.0 wt.%Si was 410.82 MPa, less than half of Somaloy 700HR 5P’s 883.03 MPa. This suggests that Fe-5.0 wt.%Si has better potential in terms of reducing mold fatigue and improving process stability during mass production.

In summary, the simulation results indicate that Somaloy 700HR 5P excels in achieving a high final density close to the target of 7.5 g/cm^3^. However, Fe-5.0 wt.%Si outperformed Somaloy 700HR 5P in density uniformity (deviation of 0.063 vs. 0.084), effective stress deviation (78.619 MPa vs. 151.705 MPa), and hydrostatic pressure deviation (410.82 MPa vs. 883.03 MPa). Notably, Fe-5.0 wt.%Si recorded about 25% lower density deviation and less than half the hydrostatic pressure deviation, which is advantageous for internal stress distribution after forming and for reducing mold fatigue. Therefore, while Somaloy 700HR 5P has strengths in achieving high final density, Fe-5.0 wt.%Si is relatively more suitable in terms of density uniformity, stress distribution stability, and mold lifespan during mass production.

However, the statistical rigor of this DOE analysis is limited because only single measurements were obtained for each Taguchi trial. As a result, full ANOVA, confidence interval estimation, and residual analysis could not be performed in this study. Instead, factor significance was inferred from S/N ratio and main effect plots, and the candidate optima were further supported by confirmation runs.

## 4. Prototype Analysis

This study includes density analysis, chemical composition analysis, microstructure analysis, and various measurements of electromagnetic properties for bulk prototypes.

### 4.1. Prototype Fabrication

**Figure 17:**
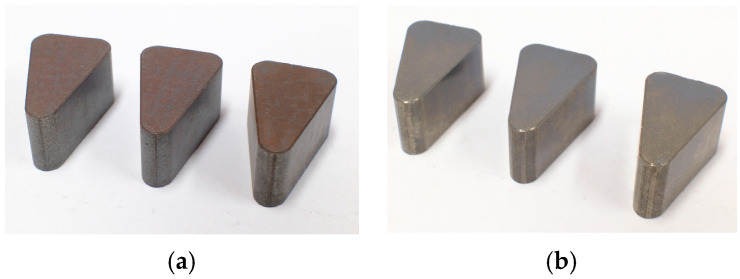
Fabricated stator core samples: (**a**) Somaloy 700HR 5P, (**b**) Fe-5.0 wt.%Si.

To verify the optimal process conditions obtained through the S/N ratio analysis of the Taguchi experimental design method, three core specimens were fabricated for each powder type, as shown in Figure 17. The density of the core specimens was measured according to the KS-D-0033 testing standard [59], which is the density test method for metal sintered bodies. The testing equipment used was the METTLER-TOLEDO XS204 (METTLER-TOLEDO, Columbus, OH, USA). Due to the porous nature of the samples, a paraffin coating was applied beforehand to enable measurement using the Archimedes method.

During density measurement, buoyancy corrections were applied using the density of distilled water at 25.0 ± 0.5 °C. A constant paraffin coating mass of 0.050 g was considered for mass/volume corrections. The measurement repeatability was ensured by *n* = 3 specimens for each powder, and the uncertainty budget included contributions from the balance resolution (0.0001 g), water temperature fluctuation (±0.5 °C), and paraffin coating correction.

For specimens made using the optimal process conditions of Somaloy 700HR 5P, the target density was 7.5 g/cm^3^. The measured densities of the fabricated specimens were 7.514, 7.507, and 7.505 g/cm^3^, respectively, with an average density of 7.509 g/cm^3^. This showed a 0.12% error compared to the target density from the compression molding analysis.

For specimens made using the optimal process conditions of Fe-5.0 wt.%Si, the target density was 7.3 g/cm^3^. The measured densities of the fabricated specimens were 7.220, 7.176, and 7.172 g/cm^3^, respectively, with an average density of 7.189 g/cm^3^. This showed a 1.52% error compared to the target density from the compression molding analysis.

Compared to the analysis results, the actual average density of the Fe-5.0 wt.%Si specimens was approximately 1.52% lower, which is attributed to differences between the idealized forming conditions assumed in the analysis and the actual process conditions. The theoretical analysis is based on precise mechanical forming under constant velocity descent conditions. However, the press used in practice is hydraulic. Although the load pressure on the forming area is initially applied based on the calculated target density, factors such as friction, powder filling uniformity, and punch alignment can cause pressures exceeding the initial value, resulting in a reaction force from the lower die that pushes the punch back. Therefore, various disturbances that may occur in the actual process are not reflected in the analysis.

Despite these differences, the density errors of approximately 0.12% and 1.52% fall well within the internationally accepted standards and industrial tolerance ranges commonly used to evaluate the agreement between numerical analysis results and experimental data. In addition, direct validation was performed by comparing simulation targets (7.5 and 7.3 g/cm^3^) with the measured averages (7.509 and 7.189 g/cm^3^, respectively). A parity plot confirmed close agreement, with R^2^ = 0.998, mean absolute percent errors of 0.12% (Somaloy 700HR 5P) and 1.52% (Fe-5.0 wt.%Si), and 95% limits of agreement derived from triplicate measurements (±0.009 g/cm^3^ and ±0.046 g/cm^3^, respectively). ASME V&V 10 (2016) presents a systematic verification procedure to ensure the validity of numerical model-based analyses, and international standards such as ISO 19364 (2016) adopt an approach where a model is considered valid if the analysis results fall within the 95% confidence interval (CI) obtained from repeated experiments [60,61].

In actual industrial and academic research, it is common to assess predictive accuracy using statistical tolerance ranges such as an average relative error of 5%, a maximum error of 10–20%, or inclusion within ±20% for 95% of cases. Accordingly, the errors of 0.12% and 1.52% confirmed in this study satisfy various international standards and statistical confidence levels, and the added statistical indicators demonstrate the quantitative reliability of the proposed numerical model experimentally.

### 4.2. Prototype Microstructure Analysis

**Figure 18:**
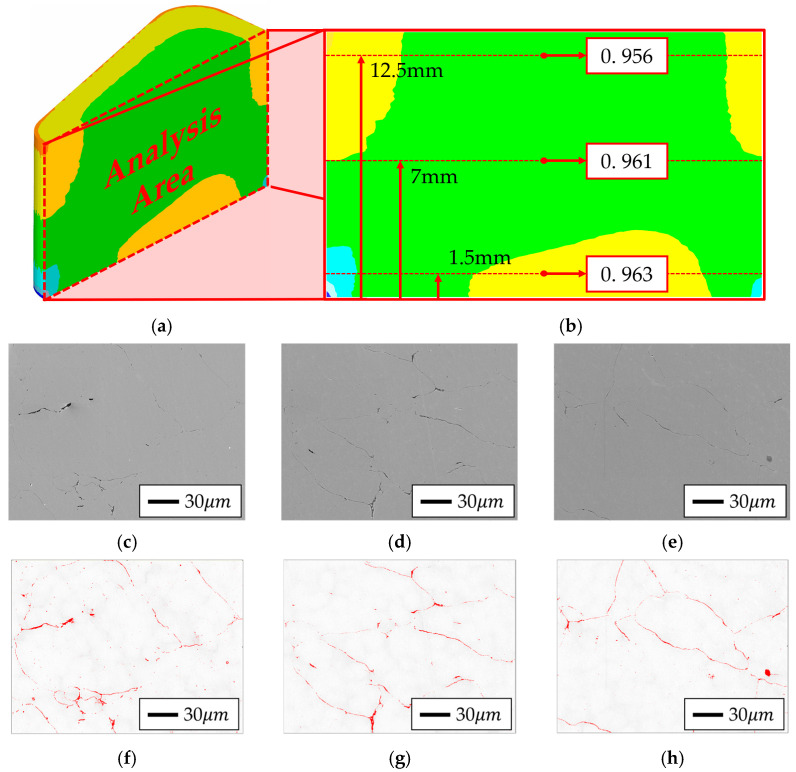
Measurement area of the Somaloy 700HR 5P core specimen and SEM results: (**a**) analysis area of specimen (**b**) relative density of stator core, (**c**) SEM of upper point, (**d**) SEM of middle point, (**e**) SEM of lower point, (**f**) upper point porosity highlight, (**g**) middle point porosity highlight, (**h**) lower point porosity highlight.

**Figure 19:**
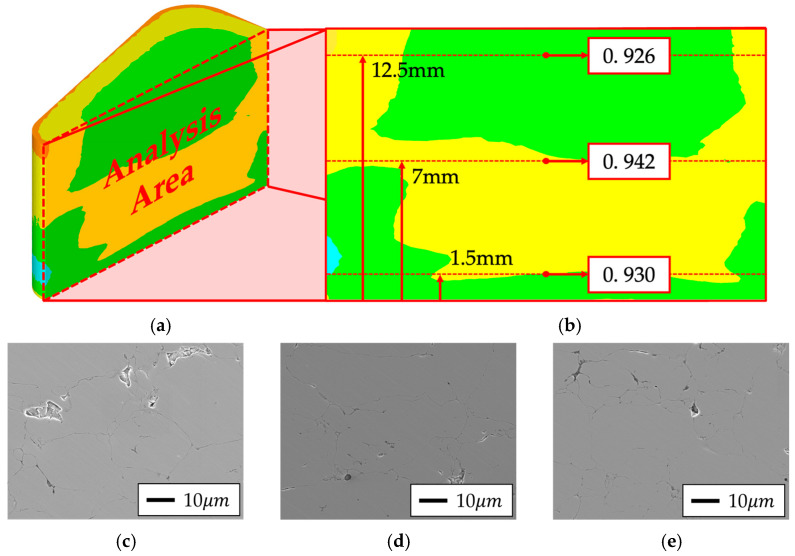

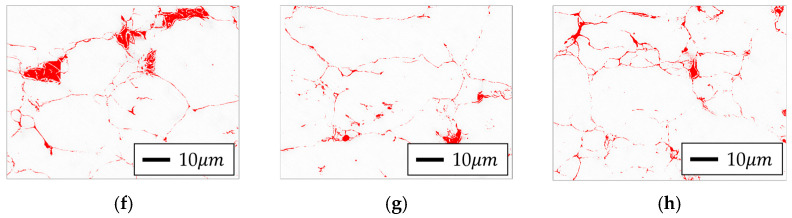
Measurement area of the Fe-5.0 wt.%Si core specimen and SEM results: (**a**) analysis area of specimen (**b**) relative density of stator core, (**c**) SEM of upper point, (**d**) SEM of middle point, (**e**) SEM of lower point, (**f**) upper point porosity highlight, (**g**) middle point porosity highlight, (**h**) lower point porosity highlight.

For the qualitative validation of the powder compaction simulation, core specimens fabricated from each powder type were sectioned, and the cross-sectional center was divided into top, middle, and bottom regions corresponding to positions 12.5 mm, 7 mm, and 1.5 mm from the bottom surface, respectively, for SEM analysis. The analysis results were compared with the compression molding simulation outcomes, and these results are presented in Figure 18 and Figure 19. Additionally, SEM and EDS analyses were conducted using a scanning electron microscope (SEM; JEOL Ltd., Tokyo, Japan) at ×500 magnification with a field size of approximately 250 × 250 µm^2^ (scale bar = 10 µm). Porosity was quantified from the acquired images using ImageJ software (National Institutes of Health, Bethesda, MD, USA; https://imagej.nih.gov/ij/, accessed on 2 August 2025) with the Otsu thresholding algorithm, and the values were reported as 2D area fractions, which are commonly regarded as proxies for 3D porosity in powder compacts. To ensure statistical reliability, 95% confidence intervals were calculated from the three regional measurements: Somaloy 700HR 5P exhibited an average porosity of 0.78% (95% CI: 0.41–1.15%), whereas Fe-5.0 wt.%Si exhibited 3.13% (95% CI: 0–6.27%). The results are summarized in Table 14.

In the case of Somaloy 700HR 5P, both the SEM results and simulation data showed the highest density at the bottom region, with calculated porosities of 0.93%, 0.79%, and 0.63% for the top, middle, and bottom sections, respectively. This trend aligns with the simulation results, supporting the reliability of the simulation. For the Fe-5.0 wt.%Si powder, the porosity calculated from the SEM images was 4.54%, 2.07%, and 2.79% for the top, middle, and bottom regions, respectively. This also matched the simulation results, confirming that the relative density was higher in the middle and bottom regions compared to the top. Notably, Somaloy 700HR 5P exhibited an average measured density of approximately 7.509 g/cm^3^, which is over 4% higher than that of Fe-5.0 wt.%Si, indicating a characteristic formation of denser particle boundaries.

**Figure 20:**
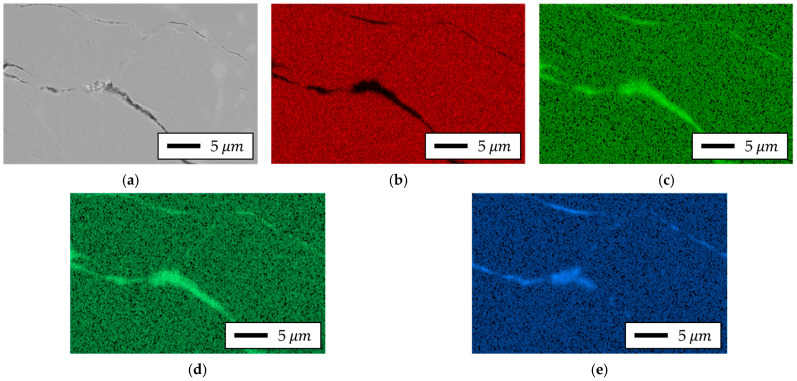
SEM-EDS image results of Somaloy 700HR 5P core specimen: (**a**) SEM-EDS image site map, (**b**) Fe, (**c**) Si, (**d**) P, (**e**) Bi.

**Figure 21:**
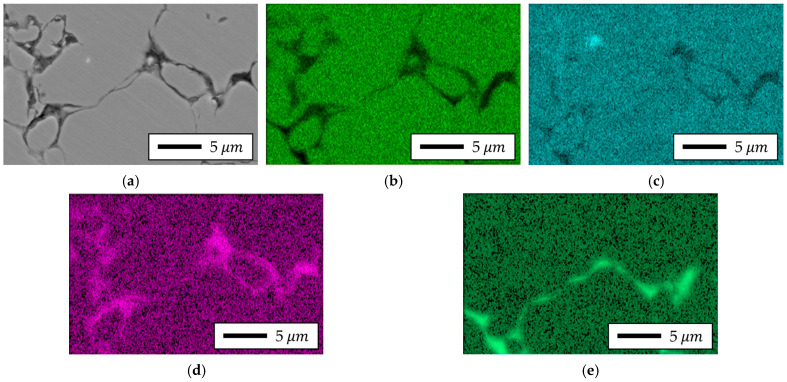
SEM-EDS image results of Fe-5.0 wt.%Si core specimen: (**a**) SEM-EDS image site map, (**b**) Fe, (**c**) Si, (**d**) P, (**e**) Mo.

To verify the chemical composition of the measurement area, EDS analysis was performed on the cross-sections of the bottom parts of specimens fabricated from each powder type. Although direct resistivity tests were not conducted in this study, the insulation state was inferred from these SEM–EDS observations in conjunction with electromagnetic performance evaluation. The results are presented in Figure 20 and Figure 21.

The analysis revealed that Somaloy 700HR 5P contained Fe, Si, P, and Bi elements, with Si, P, and Bi being particularly concentrated at the particle boundaries. Accordingly, it was confirmed that the main component of the residuals shown in the composition of Table 1 is Bi. Bi is an additive included in Höganäs’ proprietary technology, applied to the powder particle surfaces to reduce friction between particles and assist in particle insulation [62]. The distribution pattern of Bi observed in Figure 20 is consistent with the mechanism described in Höganäs patents and prior literature, which report that Bi promotes the formation and uniformity of SiO_2_ insulating layers [63]. While EDS cannot directly determine oxidation states or the continuity of thin films, the observed distributions are in agreement with these established findings. Therefore, our discussion of Bi’s role is presented as an interpretation supported by prior art, rather than a definitive conclusion drawn solely from the present EDS data.

For Fe-5.0 wt.%Si, as confirmed by the SEM results in Figure 19, a larger pore distribution and thicker insulating layer were observed compared to Somaloy 700HR 5P. The EDS results in Figure 21 showed residual phosphate components in the insulating layer, and no SiO_2_ insulating layer was formed. Additionally, a thick residual insulating composition remained along the H_3_PO_4_ layer. Furthermore, MoS_2_, used as a lubricant during the high-temperature forming process of the Fe–Si powder, acted as a factor degrading the final product’s insulating performance. Notably, EDS analysis detected Mo elements remaining at the powder boundaries, causing localized segregation of the additive, which is considered a primary cause of insulating layer non-uniformity.

Summarizing the results of the SEM and EDS analyses, both powders successfully formed the intended insulating layer; however, the Fe-5.0 wt.%Si powder exhibited a relatively higher porosity. Accordingly, the electromagnetic performance of stator core prototypes fabricated using the two powders was compared, and the effects of differences in the insulating layer and microstructure were discussed in Section 4.3.

Additionally, to monitor whether any unwanted phase transformations occurred under the high-temperature and high-pressure conditions during the core manufacturing process, X-ray diffraction (XRD) analysis was conducted. Bulk specimens were analyzed using a Rigaku SmartLab X-ray diffractometer (Rigaku Corporation, Tokyo, Japan), and the crystal structures before and after forming were compared. According to prior research, both powders were confirmed to have an Fe–Si crystal structure in the powder state, and this study aimed to verify whether this structure remained stable after forming and heat treatment.

**Figure 22:**
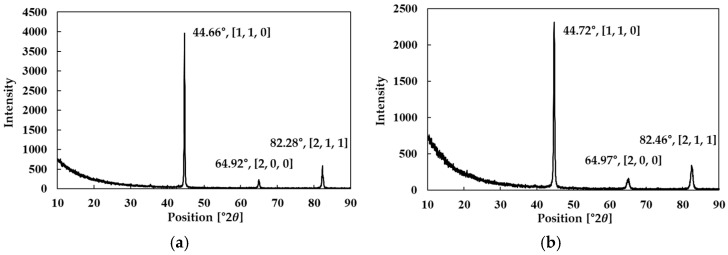
Stator core XRD results: (**a**) Somaloy 700HR 5P, (**b**) Fe-5.0 wt.%Si.

As shown in the results of Figure 22, both powders exhibited the main Fe diffraction peaks at [110], [200], and [211]. The measured 2θ positions were 44.55°, 64.97°, and 82.31° for Somaloy 700HR 5P in the powder state, and 44.66°, 64.92°, and 82.28° in the bulk state. For Fe-5.0 wt.%Si, the corresponding peaks were located at 44.77°, 65.13°, and 82.52° in the powder state, and 44.72°, 64.97°, and 82.46° in the bulk state. These slight shifts toward higher angles in Fe-5.0 wt.%Si are consistent with the effect of increased Si content on lattice contraction, as reported in Ref. [25]. In Somaloy 700HR 5P, variations in relative peak intensities are interpreted as being influenced by the insulating coating and additives. Importantly, no additional peaks corresponding to unwanted secondary phases were observed, confirming that the Fe–Si crystal structure remained stable during the forming and heat treatment processes [24].

To further substantiate these observations, the cubic lattice parameters were quantitatively calculated from the [110], [200], and [211] reflections using Cu Kα radiation (λ = 1.5406 Å). Somaloy 700HR 5P exhibited lattice parameters of 2.8670 ± 0.0002 Å in the powder state and 2.8680 ± 0.0013 Å in the stator core, while Fe-5.0 wt.%Si showed values of 2.8604 ± 0.0017 Å and 2.8622 ± 0.0003 Å, respectively. The pre–post differences remained within +0.001–0.002 Å, confirming that no unwanted phase transformation occurred during compaction and heat treatment. Moreover, Fe-5.0 wt.%Si consistently exhibited a smaller lattice parameter than Somaloy 700HR 5P, corroborating the observed higher-angle peak shift induced by the increased Si content.

### 4.3. Motor Dynamometer Test Results

**Figure 23:**
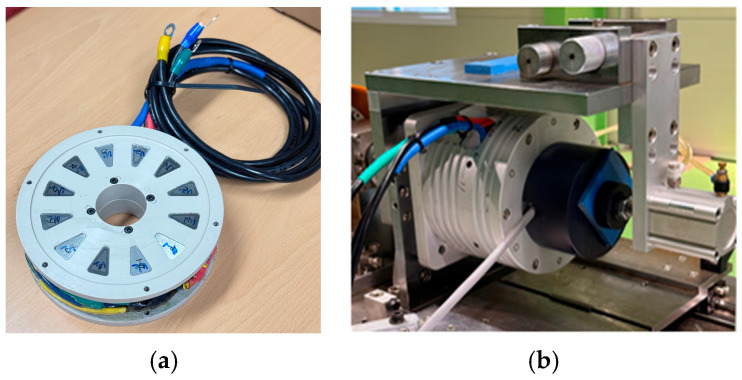
Motor equipment feature: (**a**) motor assembly, (**b**) dynamometer test equipment.

To verify the actual electromagnetic performance of the motor cores, each core was applied to an axial flux permanent magnet (AFPM) motor dynamo, and torque and output characteristics were evaluated. The AFPM motor dynamo used was designed to operate within a maximum torque of 100 N·m, a rotational speed of 10,000 rpm, and an output range up to 15 kW. Its configuration is shown in Figure 23. Performance evaluation was conducted in a domestically accredited testing environment compliant with the ISO/IEC 17025 standard [64].

The motor tests were carried out using a BLAC three-phase sinusoidal inverter (Siemens AG, Nuremberg, Germany) with a switching frequency of 10 kHz. The DC bus voltage was 72 V at the inverter input, corresponding to a phase voltage of 32 V at the motor terminals. During testing, the laboratory ambient temperature was maintained at 25–28 °C, while the motor product temperature stabilized in the 40–60 °C range. Measurements were recorded after both casing and winding resistance reached stable values within this thermal window, ensuring a steady-state condition. Torque was measured using a transducer calibrated by an ISO/IEC 17025–accredited laboratory. The calibration certificate specifies the expanded uncertainty at a 95% confidence level (k ≈ 2) as 1.7 × 10^−3^ N·m in the 10–20 N·m range and 3.0 × 10^−3^ N·m in the 20–50 N·m range. All reported torque values should therefore be interpreted within these uncertainty bounds.

To assess the performance of the Fe-5.0 wt.%Si core, a Somaloy 700HR 5P powder core was also fabricated and tested under the same motor dynamo conditions, consistent with the prior electromagnetic performance comparison based on toroidal cores. The evaluation conditions were divided into two cases: the first involved applying a current of 150 A at 1000 rpm, and the second involved applying 130 A at 2000 rpm. The torque and output results measured under each condition are summarized in Table 15.

According to the results in Table 15, under both 1000 rpm and 2000 rpm conditions, the commercial powder Somaloy 700HR 5P exhibited approximately 19–25% superior torque and output performance compared to Fe-5.0 wt.%Si. This trend contrasts with the basic electromagnetic performance evaluation based on the toroidal core, where Fe-5.0 wt.%Si showed superior results.

The primary reason for this outcome is attributed to differences in product density. The Fe-5.0 wt.%Si core demonstrated about 4% lower density compared to Somaloy 700HR 5P, which implies an approximately 4% reduction in the effective cross-sectional area through which magnetic flux can pass. Theoretically, torque is proportional to magnetic flux, and magnetic flux is defined as the product of magnetic flux density and effective cross-sectional area. Therefore, considering only the density difference, it is reasonable to expect at least a 4% decrease in performance for the Fe-5.0 wt.%Si core compared to Somaloy. In fact, the porosity calculated based on SEM analysis (Table 14) supports this density difference, and due to additional losses related to the shape, distribution, and location of pores, the effective porosity tends to appear more than four times greater.

However, the actual performance drop (approximately 19–25%) is significantly larger than the theoretical estimate (about 4%) explained by density difference alone. To further examine this effect, torque and output were normalized by the measured bulk density of each core (Appendix B, Table A3). Even after normalization, Somaloy 700HR 5P still demonstrated superior performance compared to Fe-5.0 wt.%Si, but the relative gap was reduced, confirming that differences in density and processing routes contributed substantially to the observed discrepancies. This also indicates that, in addition to product density, indirect factors arising during motor manufacturing and reassembly processes played a substantial role. Mlot et al. [65] reported that the manual manufacturing and assembly process of AFPM motors can cause instability in motor parameters, and Taran et al. [66] identified poor alignment between the stator and rotor as a key factor in motor performance degradation. Furthermore, Riquelme et al. [67] indicated that slight misalignment between the rotor and stator during motor reassembly and the resulting asymmetry in the magnetic circuit can induce cogging torque, thereby reducing performance. Synthesizing these prior research findings, it can be concluded that the performance degradation of the Fe-5.0 wt.%Si core observed in this study was influenced not only by direct factors related to density differences but also by indirect factors such as misalignment and tolerance accumulation during the assembly process.

## 5. Conclusions

In this study, Somaloy 700HR 5P and Fe-5.0 wt.%Si powders were applied to the AFPM-type motor core stator of a test low-speed electric vehicle. To this end, optimal processing conditions were derived based on simulations, and prototype samples were fabricated to comparatively evaluate the formability and performance of the prototypes.

Three process factors were selected: the corner radius of curvature of the motor core shape, forming temperature, and forming speed. A Taguchi experimental design was created accordingly. Simulations were conducted using the maximum–minimum density variation, a key characteristic that critically affects the electromagnetic performance and forming quality of the motor core.

Simulation results showed that the main factors significantly influencing the process, in order of impact, were the corner radius of curvature, forming temperature, and forming speed. The optimal processing conditions for Somaloy 700HR 5P were determined to be 3.5 mm, 50 °C, and 5 mm for each factor, respectively, while for Fe-5.0 wt.%Si, the optimal conditions were 3.5 mm, 550 °C, and 3 mm.

Using these optimal conditions, motor core prototypes were fabricated from each powder, and density measurements, microstructure analyses, and motor dynamo tests were conducted on the prototypes.

Microstructure analysis of the prototypes using SEM-EDS showed that the density distribution trends matched those predicted by the simulations, supporting the reliability of the simulation results. Additionally, XRD results of the formed prototypes for both powders were consistent with the XRD results of the powders themselves, confirming that no unnecessary phase transformations occurred during the forming process.

The motor dynamo test results were compared with the electromagnetic property evaluations conducted on the powders.

In the powder state, Fe-5.0 wt.%Si exhibited electromagnetic performance approximately 10% superior to that of Somaloy 700HR 5P. Based on this observation, Hypothesis (i) assumed that Fe-5.0 wt.%Si would also deliver superior torque and power output at the motor prototype level. However, this hypothesis was not supported: in the test motor dynamo evaluation, Fe-5.0 wt.%Si showed a performance decrease of about 19–25%.

This performance discrepancy is attributed to the following three factors:

First, the difference in target density: Fe-5.0 wt.%Si reached a target density about 4% lower than that of Somaloy 700HR 5P, which directly reduces saturation flux density and permeability as relative density increases.

Second, mechanical losses during motor assembly: Micro-stress accumulation and contact discontinuities occurring during disassembly and assembly alter the mechanical stress state within the iron core, inducing magnetic anisotropy. This leads to localized permeability reduction and increased iron losses, causing performance differences before and after assembly. Such assembly-induced loss has been reported in previous studies as a major cause of motor core performance degradation.

Third, non-uniformity of the insulation layer: Microstructure analysis and EDS results confirmed thick residual insulating compositions along the H_3_PO_4_ layer. Although MoS_2_ acted as a lubricant during forming, it contributed to reduced insulation in the final product. Notably, EDS analysis showed Mo elements remaining at powder boundaries, indicating local segregation of additives, which exacerbated the non-uniformity of insulation layer thickness and composition.

As a result, Somaloy 700HR 5P achieved excellent formability by reaching an average density close to the target value of 7.5 g/cm^3^, securing high density. However, its relative density variation was 0.084 g/cm^3^, about 25% larger than Fe-5.0 wt.%Si’s 0.063 g/cm^3^, indicating inferior internal uniformity. Hypothesis (ii) was supported: Somaloy 700HR 5P cores ultimately delivered superior torque and power output in motor dynamometer tests, consistent with their higher density and more uniform insulation.

In addition, while the Taguchi DOE framework was effective for scoping factor effects and guiding the selection of candidate optimum conditions, its statistical rigor is limited by the absence of replicated trials and ANOVA. Future work will therefore incorporate replication, ANOVA-based factor significance testing, and residual analysis to provide confidence intervals and strengthen the robustness of the optimization methodology.

The ~4% lower density of the Fe-5.0 wt.%Si cores can only explain part of the observed 19–25% performance deficit. The additional loss is plausibly due to insulation non-uniformity and assembly-induced effects, consistent with prior AFPM motor studies [65,66,67]. Therefore, considering both forming stability and electromagnetic performance comprehensively, Somaloy 700HR 5P was confirmed to be a more suitable candidate material for AFPM-type motor cores used in low-speed electric vehicles. The present findings, together with prior literature, provide robust support for this interpretation, although future modeling and dedicated experiments would be valuable to quantitatively disentangle the respective contributions.

## Figures and Tables

**Table 1:** Somaloy 700HR 5P and Fe-5.0 wt.%Si raw powder chemical composition.

Element	Somaloy 700HR 5P	Fe-5.0 wt.%Si
Fe	94.58	94.85
Si	2.74	5.02
P	1.58	-
O	0.02	0.13
Residuals	1.08	-

**Table 2:** Coating composition of SMCs powder.

Composition [wt.%]	H_3_PO_4_	PI	MoS_2_
Somaloy 700HR 5P	-	-	1.0
Fe-5.0 wt.%Si	1.0	0.5	1.0

**Table 3:** Design of experiment control factor and level of Somaloy 700HR 5P.

Description	Level		
	1	2	3
Corner radius [mm]	0.5	2.0	3.5
Molding temperature [°C]	RT	50	100
Molding Speed [mm/s]	3	5	7

**Table 4:** Design of experiment control factor and level of Fe-5.0 wt.%Si.

Description	Level		
	1	2	3
Corner radius [mm]	0.5	2.0	3.5
Molding temperature [°C]	450	550	650
Molding Speed [mm/s]	3	5	7

**Table 5:** L_9_(3^3^) orthogonal array.

Simulation No.	Factor		
	Corner Radius [mm]	Molding Temperature [°C] (Somaloy 700HR 5P/Fe-5.0 wt.%Si)	Molding Speed [mm/s]
1	0.5	RT/450	3
2	0.5	50/550	5
3	0.5	100/650	7
4	2.0	RT/450	5
5	2.0	50/550	7
6	2.0	100/650	3
7	3.5	RT/450	7
8	3.5	50/550	3
9	3.5	100/650	5

**Table 6:** Design of experiment control factor and level of Somaloy 700HR 5P and Fe-5.0 wt.%Si.

Description	Corner Radius [mm]					
	Somaloy 700HR 5P			Fe-5.0 wt.%Si		
	0.5	2.0	3.5	0.5	2.0	3.5
Target density [g/cm^3^]	7.5			7.3		
Tap density [g/cm^3^]	3.3			3.2		
Project area [mm^2^]	314.1	310.4	302.2	314.1	310.4	302.2
Powder load [g]	33.0	32.6	31.7	32.1	31.7	30.9
Compact pressure [MPa]	249.9	252.9	259.8	249.9	252.9	259.8
Initial height [mm]	31.8	31.8	31.8	31.9	31.9	31.9

**Table 7:** Number of meshes in the FEA model.

Description	Corner Radius [mm]					
	Somaloy 700HR 5P			Fe-5.0 wt.%Si		
	0.5	2.0	3.5	0.5	2.0	3.5
Numbers of elements	453,806	480,416	500,318	433,590	457,002	442,624

**Table 8:** Somlaoy 700HR 5P results for each case according to DOE.

Simulation No.	Maximum Density [g/cm^3^]	Minimum Density [g/cm^3^]	Maximum– Minimum Density [g/cm^3^]	Maximum– Minimum Effective Stress [MPa]	Average Effective Stress [MPa]	Average Mean Stress [MPa]
1	7.730	6.724	1.006	222.527	439.388	−851.575
2	7.691	6.950	0.741	190.083	430.410	−832.084
3	7.730	6.739	0.991	216.460	413.567	−802.781
4	7.706	6.911	0.803	174.936	439.918	−854.687
5	7.706	6.919	0.788	172.750	431.161	−836.287
6	7.699	6.942	0.764	168.059	413.890	−802.568
7	7.699	7.012	0.679	150.849	439.965	−851.754
8	7.699	7.004	0.686	152.697	431.175	−835.541
9	7.691	7.028	0.663	149.140	413.932	−802.298

**Table 9:** S/N ratio results of Somaloy 700HR 5P.

Simulation No.	Maximum–Minimum Density [g/cm^3^]	S/N Ratio
1	1.006	−0.05
2	0.741	2.62
3	0.991	0.06
4	0.803	1.94
5	0.788	2.06
6	0.764	2.37
7	0.679	3.32
8	0.686	3.25
9	0.663	3.56

**Table 10:** Main effects for each level of control factors results of Somaloy 700HR 5P.

Description		Corner Radius [mm]	Molding Temperature [°C]	Molding Speed [mm/s]
Level	1	0.87	1.74	1.86
	2	2.12	2.64	2.70
	3	3.38	2.00	1.81
Delta		2.50	0.90	0.89
Rank		1	2	3

**Table 11:** Fe-5.0 wt.%Si results for each case according to DOE.

Simulation No.	Maximum Density [g/cm^3^]	Minimum Density [g/cm^3^]	Maximum– Minimum Density [g/cm^3^]	Maximum– Minimum Effective Stress [MPa]	Average Effective Stress [MPa]	Average Mean Stress [MPa]
1	7.623	6.628	0.995	193.663	515.665	−762.043
2	7.739	6.392	1.348	152.207	311.947	−526.115
3	7.652	6.780	0.871	75.110	235.768	−348.241
4	7.592	6.797	0.796	162.434	521.531	−773.375
5	7.559	6.796	0.762	123.576	398.990	−588.955
6	7.595	6.799	0.797	68.409	236.327	−347.972
7	7.550	6.826	0.724	144.133	521.792	−769.770
8	7.558	7.069	0.489	78.619	399.009	−584.807
9	7.572	6.834	0.738	63.893	237.256	−350.262

**Table 12:** S/N ratio results of Fe-5.0 wt.%Si.

Simulation No.	Maximum–Minimum Density [g/cm^3^]	S/N Ratio
1	0.995	0.04
2	1.348	0.59
3	0.871	1.2
4	0.796	1.98
5	0.762	2.36
6	0.797	1.94
7	0.724	2.81
8	0.489	6.21
9	0.738	2.64

**Table 13:** Main effects for each level of control factors results of Fe-5.0 wt.%Si.

Description		Corner Radius [mm]	Molding Temperature [°C]	Molding Speed [mm/s]
Level	1	0.61	1.61	2.75
	2	2.10	3.06	1.74
	3	3.89	1.93	2.12
Delta		3.27	1.45	1.01
Rank		1	2	3

**Table 14:** Calculated porosity from SEM images.

Description	Somaloy 700HR 5P			Fe-5.0 wt.%Si		
	Upper	Middle	Lower	Upper	Middle	Lower
Porosity [%]	0.93	0.79	0.63	4.54	2.07	2.79

**Table 15:** Torque and power results as a function of RPM and input current.

RPM	1000		2000	
Input Current [A]	150		130	
Description	Somaloy 700HR 5P	Fe-5.0 wt.%Si	Somaloy 700HR 5P	Fe-5.0 wt.%Si
Torque [N·m]	41.5 ± 1.16	34.5 ± 0.21	36.4 ± 0.25	29.3 ± 0.05
Power [kW]	4.3 ± 0.12	3.6 ± 0.02	7.6 ± 0.05	6.1 ± 0.01

## Data Availability

The original contributions presented in this study are included in the article; further inquiries can be directed to the corresponding author.

## Appendix A

To verify the numerical stability of the FEM simulations, additional convergence studies were conducted for Somaloy 700HR 5P (Case 1).

Finite element models were constructed with mesh counts ranging from ~238k to ~454k elements. At the fully refined mesh of 453,806 elements, the predicted maximum relative density was 0.991018. When the mesh density exceeded ~75% of this refinement (≥342k elements), the maximum relative density stabilized within 0.9910–0.9914, corresponding to a deviation below 0.05%. The maximum effective stress values also converged to 512–515 MPa, with less than 0.5% variation. Larger deviations appeared only when the mesh count dropped below ~70% refinement (~312k elements or fewer). These results demonstrate that the FEM predictions converge once a sufficiently refined grid is applied.

Since the simulations were performed under displacement-controlled punch motion, step-size sensitivity was tested by varying increments from 0.015 mm to 0.06 mm. Across this range, the maximum relative density remained constant within ±0.0005, and the maximum effective stress varied by less than 0.4% (513–515 MPa). This confirms that the reported predictions are insensitive to displacement-step settings.

The full results of these convergence studies are summarized in Table A1 and Table A2, which provide quantitative evidence supporting the numerical stability of the FEM analyses reported in the main text.

**Table A1:** Mesh refinement convergence results for Somaloy 700HR 5P (Case 1).

Mesh Count	Percent	Max Relative Density	Max Effective Stress [MPa]
453,806	100%	0.991018	515.289
412,174	91%	0.991178	512.769
381,246	84%	0.991088	513.185
342,335	75%	0.991424	512.963
312,329	69%	0.990408	512.902
272,567	60%	0.992035	507.392
237,801	52%	0.995144	530.755

**Table A2:** Displacement-step increment sensitivity for Somaloy 700HR 5P (Case 1).

Displacement Step [mm/s]	Max Relative Density	Max Effective Stress [MPa]
0.015	0.991178	515.289
0.03	0.991391	515.471
0.045	0.990889	513.458
0.06	0.990940	513.875

## Appendix B

To reduce the influence of density differences on motor performance, torque and power were normalized by the bulk density of each core material. Table A3 presents the normalized values, expressed as average torque per unit density (Nm·cm^3^/g) and average power per unit density (kW·cm^3^/g). Although Somaloy 700HR 5P still exhibited higher values than Fe-5.0 wt.%Si, the relative performance gap was reduced after normalization. This indicates that a significant portion of the observed difference originates from density and process-related effects, rather than intrinsic material properties alone.

**Table A3:** Normalized torque and power results per density (Nm·cm^3^/g, kW·cm^3^/g).

Condition	Material	Density (g/cm^3^)	Torque (Nm)	Torque_Norm	Power (kW)	Power_Norm
1000 rpm, 150 A	Somaloy 700HR 5P	7.509	41.5	5.53	4.3	0.57
	Fe-5.0 wt.%Si	7.189	34.5	4.80	3.6	0.50
2000 rpm, 130 A	Somaloy 700HR 5P	7.509	36.4	4.85	7.6	1.01
	Fe-5.0 wt.%Si	7.189	29.3	4.07	6.1	0.85
